# Clinical Remarks on Dyspepsia

**Published:** 1889-12

**Authors:** R. C. Word

**Affiliations:** Professor of Physiology in the Southern Medical College, Atlanta, Ga.


### Southern Medical Record.

A MONTHLY JOURNAL OF PRACTICAL MEDICINE.

☞ All communications must be addressed to R. C. WORD, Managing Editor.

"*Quicquid Præcipies Esto Brevis.*"

VOL. XIX ATLANTA, GA., DECEMBER, 1889. NO. 12

### ORIGINAL AND SELECTED ARTICLES.

### CLINICAL REMARKS ON DYSPEPSIA.

BY R. C. WORD, M. D.,

Professor of Physiology in the Southern Medical College, Atlanta, Ga.

(*Republished by request of the Class.*)

GENTLEMEN: The word dyspepsia is from the Greek *dus*, difficulty, and *pepto*, to concoct or digest. The word indigestion has the same signification. It presents itself to the practitioner in numerous phases. To understand, or to distinguish the varied forms of the affection, so as to apply the proper treatment, requires a thorough knowledge of the physiological processes involved in healthful, or normal, digestion. Unfortunately, many medical practitioners give no attention to the scientific aspects of the subject, and use the same treatment indiscriminately for every phaze of the disease. The result is, of course, failure in a large majority of cases.

When consulted in a case of indigestion, the practitioner should carefully investigate the symptoms, in order to determine the particular link, or links, in the complicated chain of the digestive apparatus which is at fault, and direct his treatment to the special points of trouble.

We can not here enter into detail as to the minute chemical and physiological changes which are wrought upon alimentary substances in the normal processes of digestion, but will briefly glance at the leading and more essential features. And first, it should be borne in mind that the food must be thoroughly masticated and mixed with saliva, by which it is rendered alkaline and the starchy portions of the food converted into sugar, by which it is made soluble: the organic ferment of the saliva, known as ptyalin, is the principal agent in this process.

Next, the contents of the stomach are made acid by the gastric juice, the ferment of which, *pepsin*, dissolves the connective tissue of meat foods, converting proteids into peptones, and turning loose the fats. A pulpy mass called chyme is formed, made up of liquid fats, peptones and remains of starch unconverted. These pass into the duodenum for further action. Here the bile, the intestinal juices and the pancreatic juices are poured in upon the mixture. The mass is changed from an acid into an alkaline state; the fats are emulsified and made soluble by the bile and pancreatic juice, the latter serving, also, to change any undissolved portions of starch into sugar. The peptones are taken up by the portal vein, and the emulsified fats by the lacteals. Peptones formed in the stomach are absorbed by the capillary blood-vessels and villi, and, through the portal vein, find their way to the liver. In the liver they are converted into glycogen, urea and kreatin, or, may be, reconverted into albumen for purposes of nutrition. The dextrine, or sugar, produced in the stomach from starch by the action of the saliva, and that formed in the duodenum by the action of the pancreatic juice, also enter the portal vein, and thus reach the liver, where it is changed, or fitted for entrance into the general circulation through the hepatic vein, and is oxidized in the lungs, producing animal heat.

Any excess of albuminoids and sugars not appropriated are thrown off through the kidneys and skin as urea, lithic acid, etc. It is essential to the proper action of these processes that the peristalsis of the entire alimentary canal should be in vigorous, uninterrupted action, so that the movements of the stomach may be complete, the necessary downward movement of alimentary substances be uniform, and that excrementatious matters be discharged by defecation regularly and daily performed.

From this brief reference to the several stages of the process of digestion, it is evident that dyspepsia may result from the imperfect performance of any one of the series of the several functions named, or that two or more of the separate stages in the process might conjoin as causes of the trouble, and that the degree of suffering and the phases of disorder presented would vary accordingly. Hence, the forms of indigestion which the physician is called upon to treat are exceedingly numerous, and when considered in connection with idiosyncracies, peculiar temperaments and the reflex influences which radiate from and upon the stomach, they may be said to embrace a large proportion of the ills that flesh is heir to.

We have said that starch is converted into sugar, or dextrine, through the agency of a ferment contained in the saliva. Chemically speaking, it is made soluble by hydration, a molecule of water being added to it. For the solution of starch, as we have it in potatoes, flour, corn, tapioca, etc., thorough mastication and insalivation is necessary. Mastication serves to break down and disintegrate the particles of food. The grinding of grain before cooking facilitates the mastication, and is important. So the cooking breaks down the starchy particles and gives better access to the salivary secretion. To this part of the process, good teeth and slow chewing are important. In some cases of indigestion, it may be well to inquire as to whether the saliva is sufficiently abundant and of normal quality. This is a point which, so far as we know, has not been mentioned in the treatment as given by the authorities. The normal salivary secretion is alkaline; and yet, in some instances, it has been found to give an acid reaction, in which case we might expect to find the starchy portions of the food undigested, giving rise to acid eructations, the evolution of gasses, flatulence, distension of the stomach, etc. Like symptoms would result if there were a deficiency of the quantity of saliva, whether caused by insufficient secretion, by imperfect mastication, or by hurried eating. For immediate relief in such conditions, alkalies are used. From a half to a teaspoonful of the bicarbonate of soda, or a teaspoonful of calcined magnesia, will almost invariably give present relief, but to prevent its recurrence the patient must be instructed to eat slowly and chew well. As a remedy to improve the salivary secretion, the bitter tonics, with alkalies, should be used.

| | |
|---|---|
| ℞ Compound tinc. gentian<br>Syr. rhubarb aromat | } ää ℥ iv. |
| Bi carb. soda | ʒ ij. |

M. Tablespoonful before meals.

Fast eating is one of the evils of the present age, and particularly so with the American people, whose habits of running to and fro upon railroads, in the constant rush of business, gives but little time to eat; and the mental care and worry of life, by interfering with the proper force and distribution of nervous influence, impairs the secretions and interferes with the digestive processes. The want of sleep, also, and the irregularity as to the time of eating, are among the causes which are fast making a nation of dyspeptics. Mental influence of a depressing character, or too close application to study, tends to impair digestion by drawing off nerve force from the stomach, and by deranging the salivary and gastric secretions. Who has not observed in himself the power of a sudden mental shock in destroying the appetite? Bad news received while at a meal, or the occurrence of violent anger, will almost invariably arrest the secretion of gastric juice, and has been often known to develop a violent attack of flatulent colic.

Where insalivation is at fault, from whatever cause, the digestion is impaired by the non-conversion of starch into soluble sugar, as we have said. Slow eating and thorough mastication, the use of but little water while eating, with cheerful surroundings, are the means recommended to relieve indigestion from this cause. Maltine and malt extracts containing distase—which, like ptyalin, has the property of converting starch into dextrine and into maltose—may be administered with good results in these cases, especially in the cases of children in whom the salivary secretion is often imperfect or deficient.

Any suppression or checking of the secretion of the gastric juice must derange digestion by interfering with the conversion of albuminoids into peptones. As a result, fermentative and putrefactive action of the stomach. Chyme is imperfectly formed, and undissolved portions of food finding their way into the pyloric orifice give rise to pain and sometimes to cramp colic, or being hurried through the small intestine, may cause diarrhœa or cholera morbus.

There may be excessive, or hypersecretion of gastric juice, in which case we have a burning sensation somewhat more intense than the ordinary heartburn resulting from the excessive acidity of the secretions of the stomach. Where the gastric juice is excessive, there is not so much flatulence, but more pain. In some instances the gastric secretion is thrown into the empty stomach, producing pain and a burning sensation which may be relieved by food. If not so relieved, or by a full dose of soda or other alkali, vomiting will result, and an intensely sour fluid containing hydrochloric acid, will be ejected, excoriating the throat and putting, as we say, the teeth "on edge." In cases of excessive secretion, let food be taken often and regularly and alkalies administered, and let the bowels be kept open.

In cases where the gastric juice is deficient, there is a degree of flatulence, fetid gases are generated and the eructations indicate the presence of sulphuretted hydrogen.

For this latter condition the acid phosphates have been prescribed with benefit, or the nitro-muriatic acid may be given. Theoretically, pepsin, ingluven, pancreopepsin and lactopeptin, with antiferments and antiseptics are indicated.

In cases where heartburn results from simple acid fermentation, it does not, as a rule, come on so soon after eating as in the case of hyper-secretion. There is more gas and flatulence, not so much pain, though there may be fulness and oppression, and sometimes globus hystericus, palpitation of the heart and oppression of spirits. Vomiting rarely occurs, though eructations of acid and belching up of the food frequently take place.

Too much sugar in the stomach may result from eating too much of the starchy class of food, the function of insalivation being well performed, or from an excessive use of syrups and sweets. The result is acid fermentation, headache, vertigo and often diarrhœa.

An excess of peptones, resulting from the digestion of an excess of albuminoids, meats, etc., is a frequent source of trouble in the liver, as here these peptones are converted into glycogen and urea, and any excess of urea in the blood must lead to neuralgic and gouty troubles; especially if the skin and kidneys are impaired in action, as it is through these emunctories that the economy seeks to relieve itself of these poisons. In cases wherein the liver is thus burdened, there is a tendency to congestion of the organ. and to that bilious or disordered state of the alimentary canal which leads to nervous and sick headach, migraine, etc. During a surcharge of this kind the secretion of bile is interrupted or impaired, or the organ may throw off an acrid, vitiated bile, which, by reason of a loaded or conjested state of the duodenum, may regurgitate into the stomach, giving rise to nausea, retching and vomiting, and to all those distressing symptoms which are so often met with in fashionable society from over-eating. Here, after Nature has prepared the way by emptying the stomach and duodenum, relief from the headache may be obtained by morphine hypodermically administered and keeping the patient quiet and without anything upon the stomach for a day or two. In most cases it is well to give a grain of saccharated calomel or a small blue pill at the time of using the hypodermic injection, which, acting next day, leaves the liver in a condition more favorable to the recovery of the patient.

In the milder cases of this kind, and in ordinary nervous headache, where the nausea does not prevent the use of remedies by the stomach, the following prescription I have found very useful:

| | | |
|---|---|---|
| ℞ | Bromide of ammonium | ʒ iij. |
| | Deodorized tinc. opium | ʒ ijss. |
| | Camphor water | ℥ ij. |

M. Dose, one teaspoonful every two hours until relieved.

The functions of the duodenum are highly important in the process of digestion. Chyle is here first formed and extracted from chyme; here the bile and pancreatic juices accomplish their work, fats are emulsified and the remnants of starch unconverted in the stomach are changed into soluble dextrine.

The pancreatic juice and the bile, one or both, may be deficient in quantity and perverted in quality. To facilitate the efficient performance of their functions, the remedies mostly relied upon are those that act upon the duodenum. Of these, blue pill or calomel alone, or combined with small doses of ipecac or colocynth, will be found useful. The mercurials seem to exert a specific action on the liver, as indeed, upon the glandular system generally.

Ipecac in small doses, alone or combined with other agents, acts favorably upon the duodenum and upon a torpid or congested liver.

Ipecac, grs. ¼, with calomel, grs. ¼, taken a few times as preliminary to the use of tonics, will usually prove beneficial.

| | | |
|---|---|---|
| ℞ | Com. ext. colocynth } | ää grs. v. |
| | Blue pill } | |
| | Ipecac | grs. ½. |

M. One pill as a bilious cathartic. Useful, also, in duodenal congestion.

| | | |
|---|---|---|
| ℞ | Blue pill | grs. lx. |
| | Podophyllin | grs. viij. |

M. Pills No. xxxii, one to be taken occasionally. Useful in torpor of the liver and duodenum.

The drugs just mentioned, as also senna, euonymus and podophyllin, act upon the duodenum in such manner as to increase its peristalsis, stimulate its secretions, relieve hyperæmic or congested conditions of the part, and open and free the pancreatic and bile ducts to an easy and ready discharge of their contents. That calomel has this effect upon the duodenum is abundantly confirmed by experience.

The points we have referred to embrace the leading or more prominent functions connected with digestion, and should be carefully studied as landmarks for guidance. Upon careful investigation every case of indigestion you may be called to treat will be found to involve one or more of the elements to which I have referred. In the matter of treatment we have only glanced at the general principles, and must leave you to judge of each individual case as it presents itself, directing your treatment accordingly.

To one other point I again recur, and that is imperfect or irregular peristalsis. Herein is included constipation, which is a frequent trouble, and is, indeed, the sole cause of many cases of indigestion ; especially frequent in persons of studious and sedentary habits. *Nux vomica*, having the property of stimulating peristalsis, is a remedy of great utility in the treatment of dyspeptic cases. It is indicated in every case attended with deficient peristaltic action, or constipation in any degree. In dyspepsia from simple atony of the stomach, this remedy is indicated, and in every phase of the disease attended with torpor of the bowels it should be prescribed. In such cases the breath is bad and the tongue is covered with a white fur and presents a somewhat large and flabby appearance.

Here the nux may be used. The formula containing this article may be varied or combined to suit the special conditions of the case.

| | | |
|---|---|---|
| ℞ | Ext. nux vomica | grs. ¼. |
| | Aloes soc. | grs. ij. |

At bed time as a laxative.

This is especially adapted to torpor of the colon or rectum.

| | | |
|---|---|---|
| ℞ | Aloes soc. | grs. xl. |
| | Ext. hyoscyami | grs. xx. |
| | Ext. nux vomica | grs. v. |
| | Ipecac | grs. v. |

M. Make pills No. xx. Take one at bed time. Anodyne at night, laxative at morning.

In debilitated subjects, especially in those of an anæmic tendency, this remedy, as found in the elixir phosphate of iron, quinine and strychnia, will, in most instances, give good results in toning up the system, improving the blood and encouraging regular action of the bowels. The same in pill form is often more efficient in action and acceptable to the patient.

| | | |
|---|---|---|
| ℞ | Pyrophosphate of iron | } āā ʒ j. |
| | Sulph. quinine | } |
| | Strychnia | grs. I. |
| | Ext. quassia | q. s. |

M. Make pill No. lx. S. Take one before each meal.

The following is excellent :

| | | |
|---|---|---|
| ℞ | Asafœtida | grs. xl. |
| | Ext. nux vomica | grs. x. |
| | Iron by hydrogen | grs. xl. |
| | Ext. quassia | q. s. |

M. Make pills No. xl. Take one three times a day.

Tonic, nervine, laxative; useful in nervous dyspepsia, attended with flatulence and constipation, and especially adapted to anæmic and hysterical females.

*Podophyllin* in minute doses, as an alterative and peristaltic persuader, I have found a useful agent in torpid liver, and as a remedy for constipation, not only in dyspepsia, but in all affections where constipation requires to be corrected. The one-twentieth or the one-fortieth of a grain repeated at intervals of four to six hours will be found an efficient alterative in such cases. I have been accustomed to use for this purpose the parvules of Wm. R. Warner & Co., containing each the one-fortieth of a grain of podophyllin. One or two of the parvules, taken three times a day, will, in many cases, relieve constipation and cause a regular and easy evacuation of the bowels daily, at the same time exerting a beneficial influence upon the liver and the alimentary secretions. In cases somewhat obstinate, demanding stronger action, one or two of his aloin parvules, taken with the podophyllin, will suffice to give a mild evacuation. When a safe and efficient purgative is demanded, three to five of the aloin parvules may be given.

Touching the diet of dyspeptics, I will not attempt any specific rules. The general principles which I have given will, in a measure, furnish a guide to the practitioner.

If insalivation is at fault, be careful as to the use of starchy articles of food. If the gastric juice is deficient, be careful in the use of meats and albuminoids. In general, a spare diet at long intervals, moderate exercise in the open air in agreeable occupation, or traveling, tent life, and a pleasing diversion of mind will benefit the dyspeptic, and will be sufficient, in many instances, to effect a cure.

---

In the *Pacific Medical Journal* for July, Dr. J. H. Wythe presents a case of aortic insufficiency in which the compensatory hypertrophy of the left ventricle was so great that the heart, on autopsy, weighed forty ounces, and some of the columnæ of the left ventricle had thickened to the size of the little finger. The case had presented marked symptoms of angina pectoris.

